# Therapist adherence in the strong without anorexia nervosa (SWAN) study: A randomized controlled trial of three treatments for adults with anorexia nervosa

**DOI:** 10.1002/eat.22455

**Published:** 2015-11-18

**Authors:** Louise J Andony, Elaine Tay, Karina L Allen, Tracey D Wade, Phillipa Hay, Stephen Touyz, Virginia VW McIntosh, Janet Treasure, Ulrike H Schmidt, Christopher G Fairburn, David M Erceg-Hurn, Anthea Fursland, Ross D Crosby, Susan M Byrne

**Affiliations:** 1 School of Psychology, University of Western AustraliaPerth, Western Australia; 2 Centre for Clinical InterventionsPerth, Western Australia; 3 School of Psychology, Flinders UniversityAdelaide, South Australia; 4 School of Medicine, University of Western SydneySydney, New South Wales; 5 School of Psychology, University of SydneySydney, New South Wales; 6 Department of Psychological Medicine, University of OtagoDunedin, New Zealand; 7 Psychological Medicine, King’s College LondonLondon, United Kingdom; 8 Department of Psychiatry, University of OxfordOxford, United Kingdom; 9 Neuropsychiatric Research InstituteFargo, North Dakota

**Keywords:** therapist adherence, anorexia nervosa, CBT-E, MANTRA, SSCM, randomized controlled trial

## Abstract

**Objective:**

To develop a psychotherapy rating scale to measure therapist adherence in the Strong Without Anorexia Nervosa (SWAN) study, a multi-center randomized controlled trial comparing three different psychological treatments for adults with anorexia nervosa. The three treatments under investigation were Enhanced Cognitive Behavioural Therapy (CBT-E), the Maudsley Anorexia Nervosa Treatment for Adults (MANTRA), and Specialist Supportive Clinical Management (SSCM).

**Method:**

The SWAN Psychotherapy Rating Scale (SWAN-PRS) was developed, after consultation with the developers of the treatments, and refined. Using the SWAN-PRS, two independent raters initially rated 48 audiotapes of treatment sessions to yield inter-rater reliability data. One rater proceeded to rate a total of 98 audiotapes from 64 trial participants.

**Results:**

The SWAN-PRS demonstrated sound psychometric properties, and was considered a reliable measure of therapist adherence. The three treatments were highly distinguishable by independent raters, with therapists demonstrating significantly more behaviors consistent with the actual allocated treatment compared to the other two treatment modalities. There were no significant site differences in therapist adherence observed.

**Discussion:**

The findings provide support for the internal validity of the SWAN study. The SWAN-PRS was deemed suitable for use in other trials involving CBT-E, MANTRA, or SSCM. © 2015 The Authors. International Journal of Eating Disorders Published by Wiley Periodicals, Inc. (Int J Eat Disord 2015; 48:1170–1175)

## Introduction

In clinical trials, measuring the extent to which therapists implement treatments in accordance with their respective protocols is essential, in order for conclusions regarding treatment efficacy to be confidently determined.^1^,^2^ Assessing therapist adherence also provides an indication whether treatments under investigation can be differentiated.^2^,^3^ Therapist adherence is commonly measured by reviewing recordings of therapy sessions, and rating whether core treatment components are observed using a suitable rating scale. These rating scales also tend to include items that measure non-specific factors, such as therapist empathy.^1^,^4^,^5^ A well-known adherence scale is the Collaborative Study Psychotherapy Rating Scale (CSPRS), which was designed to assess whether therapists involved in the National Institute of Mental Health Treatment of Depression Collaborative Research Program adhered to the four treatments being compared (Unpublished manuscript).^6^,^7^

Because there are no “gold standard” treatments for adults with anorexia nervosa (AN), it is particularly important to ensure treatments involved in randomized clinical trials (RCT) are implemented in line with their specifications. Only one study has examined therapist adherence in a RCT of treatments for AN.^8^,^9^ McIntosh et al.^9^ used a modified version of the CSPRS (CSPRS-AN) to investigate adherence to, and differentiation between Cognitive Behavioural Therapy, Interpersonal Psychotherapy and Specialist Supportive Clinical management (SSCM). Results indicated that the 90-item CSPRS-AN was able to differentiate treatments reliably, and no differences in therapist adherence were found.

The current study examined therapist adherence in the Strong Without Anorexia Nervosa (SWAN) study, a multi-center RCT comparing three different psychological treatments for adults with AN^10^: Enhanced Cognitive Behavioural Therapy (CBT-E)^11^; Maudsley Model of Anorexia Nervosa Treatment for Adults (MANTRA)^12^,^13^; and SSCM.^14^ The aims were to (1) develop and test a measure of therapist adherence for use in the SWAN study, (2) assess therapist adherence to the treatments under investigation, and (3) examine inter-site differences in therapist adherence.

## Method

### Participants

Participants in the SWAN study were 120 individuals (97.5% female) recruited at three Australian sites: Perth (*n* = 80); Adelaide (*n* = 21), and Sydney (*n* = 19). Participants in the current study were 64 females drawn from this broader participant pool (Age: *M* = 26.72, SD = 10.21). Ethics approval was obtained and all participants provided informed consent.

Inclusion criteria for the SWAN study were: body mass index (BMI) ≥14.0 and ≤18.5; aged 17 years and over; and meeting diagnostic criteria A and B for AN in DSM-IV-TR.^15^ Exclusion criteria were severe medical or suicidal risk, inability to complete full treatment course, and current use of olanzapine or other active psychotherapy. Participants were randomized to one of the three treatments. Number of treatment sessions allocated was titrated according to BMI (40 sessions for BMI: <16; 30 sessions for BMI: 16–17.5; 25 sessions for BMI: 17.5–18.5).

*Therapists* were psychologists (*n =* 8) with at least two years of experience in delivering specialized psychological treatments for eating disorders. Therapists delivered all three treatments and received training by the treatment developers prior to study commencement. Therapists attended 2 h of supervision weekly with chief investigators (SB, TW, PH).

*Raters* were two female postgraduate clinical psychology students (ET, LA). Raters received 15 h of training in the use of the SWAN Psychotherapy Rating Scale to ensure consistent interpretation of scale items.

### Measures

#### Audiotapes

Ninety-eight audiotapes of full therapy sessions were randomly selected by the trial co-ordinator (KA; Perth = 72 audiotapes, Adelaide = 9; Sydney = 17). Audiotapes were selected from the early–mid treatment phase and the mid–late treatment phase. One audiotape was excluded due to crisis management circumstances. Audiotapes were selected to ensure that all treatments were comparably represented (CBT-E = 30 audiotapes; MANTRA = 32; SSCM = 35).

#### SWAN Psychotherapy Rating Scales (SWAN-PRS)

The SWAN-PRS was developed by adapting the CSPRS-AN^9^ to form a 52-item measure with 15 CBT-E specific items; 10 MANTRA specific items; 4 SSCM specific items; 11 overlap items; and 12 Non-Specific items. Items are rated on a Likert-type scale ranging from 1 (not at all) to 7 (extensively). Higher scores indicate greater adherence to the specified therapist behavior.

### Procedure

Both raters co-rated 48 audiotapes to provide a measure of inter-rater reliability before Rater 1 proceeded to rate the remaining 50 audiotapes independently. Raters were blind to treatment type.

## Results

### Factor Analysis

Principal Axis Factor Analysis with oblique rotation was used to examine the underlying factor structure of the SWAN-PRS to allow robust, treatment-specific subscales to be determined. The analysis yielded a final SWAN-PRS that included 8 CBT-E items, 9 MANTRA items, 4 SSCM items, and 12 Non-Specific Factor items. See Appendices A and B for further details pertaining to factor analyses and inter-rater reliability.

### Agreement Between Treatment Allocation and Treatment Classification

Mean subscale scores for CBT-E, MANTRA, and SSCM were calculated for all audiotapes. The highest subscale score for each audiotape was used to determine *treatment classification* (e.g., audiotapes were classified as SSCM, if the SSCM subscale score was greater than the MANTRA or CBT-E subscale scores). Eighty-six percent (*N* = 83/97) of total audiotapes were correctly classified as the treatment delivered. For CBT-E (*n =* 30), 90.0% tapes were classified accurately, while 6.7% were misclassified as MANTRA, and 3.3% as SSCM. For MANTRA (*n =* 32), 81.2% were correctly classified, while 6.3% were misclassified as CBT-E and 12.5% as SSCM. For SSCM (*n =* 35), 85.7% were accurately classified, while 8.6% were misclassified as MANTRA and 5.7% as CBT-E. There were no significant differences between treatments with regards to agreement between treatment allocation and treatment classification, *χ*^2^ (df = 2) = 0.961, *p* = .619.

**Figure 1:**
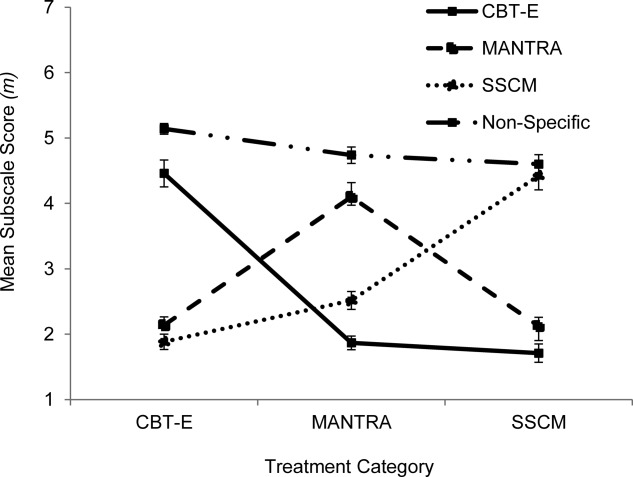
Mean treatment-specific and non-specific subscale scores for each allocated treatment modality. Error bars represent the standard error of the mean. Note: CBT-E = Enhanced Cognitive Behavioural Therapy; MANTRA = Maudsley Anorexia Nervosa Treatment for Adults; SSCM = Specialist Supportive Clinical Management.

One-way ANOVA was used to compare subscale scores for each treatment category. There was a significant overall difference found between allocated treatment groups on the CBT-E (*F*(2) = 97.68, *p* < .001), MANTRA (*F*(2) = 41.50, *p* = .001), SSCM (*F*(2) = 57.68, *p* < .001), and Non-specific (*F*(2) = 7.58, *p* < .05) subscales. Post hoc analyses (Games-Howell tests) indicated that (i) the CBT-E subscale score was significantly higher for CBT-E than for MANTRA or SSCM (*p′*s < .001), with no significant difference between MANTRA and SSCM (*p* = .754); (ii) the MANTRA subscale score was significantly higher for MANTRA than for CBT-E and SSCM (*p′*s < .001), with no significant difference between CBT-E and SSCM ( = .99); (iii) the SSCM subscale score was significantly higher for SSCM than for CBT-E and MANTRA (*p′*s < .001) and the MANTRA score was significantly higher than the CBT-E score (*p* < .05); (iv) the Non-Specifics subscale score was significantly higher for CBT-E (*M* = 5.14, SD = 0.44) than for SSCM (*p* < .01) and MANTRA (*p* < .05) but there was no significant difference between MANTRA and SSCM (*p* = .76). These results are illustrated in **Figure** 1.

### Site Differences

Fisher’s Exact tests indicated that there were no significant differences in overall adherence between sites (*p* = .52), nor for CBT-E (*p* = .99), MANTRA (*p* = .36) or SSCM, (*p* = .61), when considered separately. Kruskal–Wallis one-way ANOVA was used to compare subscale scores for each treatment according to site. There were no significant differences between sites in: CBT-E subscale scores for CBT-E therapy sessions, *F*(2) = 0.37, *p =* .691; MANTRA subscale scores for MANTRA sessions, *F*(2) = 0.36, *p =* .70; or SSCM subscale scores for SSCM sessions *F*(2) = 0.37, *p =* .69. Subscale scores were appropriately higher for the allocated treatment compared to the other treatments. There were also no significant differences between sites in Non-Specifics subscale scores *F*(2) = 0.26, *p =* .08.

## Discussion

The CSPRS-AN^9^ was successfully adapted to form a therapist adherence measure that can reliably distinguish between, and measure therapist adherence to, CBT-E, MANTRA, and SSCM. Using the SWAN-PRS, we were able to demonstrate very high agreement between actual treatment allocation and treatment classification in the SWAN study, with 85.6% of audiotapes being correctly classified by independent raters. Further, SWAN-PRS ratings demonstrated significantly higher mean therapy-specific subscale scores appropriate to the actual allocated treatment modality. We were also able to demonstrate that there were no inter-site differences in therapist adherence with regards to either treatment classification or mean subscale ratings for CBT-E, MANTRA, SSCM or the Non-Specifics subscale. These findings confirm that therapists adhered strongly to treatment protocols; that the three treatment modalities could be reliably distinguished; and that therapist adherence was not influenced by treatment site. This not only provides evidence regarding the relative ease with which treatments can be disseminated across sites, but also provides evidence for the internal validity of the study.

Limitations of the present study included the use of only two raters to establish inter-rater reliability, the use of data generated by only one rater for the remaining analyses, and the uneven distribution of audiotapes available for each treatment site. In addition, whilst the SWAN-PRS was developed to measure the extent to which therapists demonstrated behaviors consistent with treatment protocols, it did not measure therapist competence, or the extent to which therapists delivered treatments according to an acceptable standard.^3^,^11^ Therapist competence is another important aspect of treatment integrity in outcome research and should form the focus of future research.

In summary, measuring therapist adherence in the SWAN study was essential to provide evidence of the scientific quality of the trial. The rigorous development and piloting process involved in the development of the SWAN-PRS resulted in a reliable measure of therapist adherence to the three treatment modalities under investigation. The SWAN-PRS can now be used to demonstrate therapist adherence in other contexts involving these treatments. It could also generate useful information to assist with therapist training.

## Appendix A Interrater Reliability

Intra-class coefficients^16^ were used to assess inter-rater reliability for CBT-E, MANTRA, SSCM, and Non-Specific subscale ratings. Cohen’s Kappa was used to assess inter-rater reliability for treatment classification, with the strength of the Kappa statistic defined according to the following criteria: Poor (<0.20), fair (0.21–0.40), moderate (0.41–0.60), substantial (0.61–0.80), and very high agreement (0.81–1.00).^17^,^18^

Intraclass coefficients yielded a high level of agreement between raters according to Cohen’s Kappa for the CBT-E (0.83), MANTRA (0.84), SSCM (0.79), and Non-Specific (0.68) subscales. Very high agreement between raters (Kappa = 0.81, *p* < .001) was established for classification of treatments using the SWAN-PRS. Overall, 87.5% (*n* = 42/48) of audiotapes were classified as the same treatment by both raters with high inter-rater reliability demonstrated for CBT (91.67%), MANTRA (88.23%), and SSCM (84.21%), when considered separately.

## Appendix B Factor Analysis of the SWAN-PRS

An exploratory factor analysis was used to ascertain the underlying factor structure of the SWAN-PRS. Overlap items and Non-Specific items were excluded from the exploratory factor analysis, in order to obtain a factor structure reflective of the “pure” treatment components. Three factors were initially requested, as the items were specifically designed to measure three treatment-specific subscales. However, given the position of SSCM as an atheoretical treatment with broad underpinnings found in clinical management and supportive psychotherapy, it was expected that the SSCM items may load across all factors.^14^

The item inter-correlation matrix was examined and items that correlated weakly (<0.40) with other items on the same subscale were removed. The remaining items were entered into a Principal Axis Factor analysis with oblique rotation, with three factors requested. The three factors explained 60.1% of the variance, although the third factor was weak (Eigenvalues 6.98, 4.94, and 1.23). The first two factors were clearly representative of the CBT-E and MANTRA subscales. As expected, the four SSCM items tended to cross-load across the first two factors rather than loading strongly on a third factor.

Examination of the item inter-correlation matrix showed that while the four SSCM items were all highly and positively correlated (average correlation = .56), they were negatively and weakly correlated with the CBT-E and MANTRA items (all < 36). Therefore it was decided to remove the SSCM items and re-run the factor analysis with a two factor solution requested. Fifty-six percent of the variance was explained, with 8 items loading strongly (>.5) onto the CBT-E factor (eigenvalue 3.28), and nine items loading (>.5) onto the MANTRA factor (eigenvalue 3.28). Table B1 displays the items, factor loadings, and communalities for the rotated factors. A reliability analysis was used to assess internal consistency, and results revealed an acceptable Cronbach’s alpha for the CBT-E (*α* = 0.89) and MANTRA (*α* = 0.91) factors. An acceptable Cronbach’s alpha^18^ was also found for the four-item SSCM subscale (*α* = 0.76). The SSCM and Non-Specific factor subscale items are listed in Table B2.

**B1:** Factor loadings for the rotated factors: SWAN-PRS treatment-specific subscales

Item	Subscale	Factor Loading		Communality
		1	2	
Did the therapist make use of a collaborative, active, empathic stance, to encourage the client to make changes?	MANTRA	0.90	0.08	0.74
Did the therapist and client: (i) collaboratively develop a formulation OR refer to such a formulation as previously developed with the client, making reference to thinking styles, social and emotional functioning, interpersonal factors, and the valued nature of Anorexia OR (ii) explore maintaining mechanisms (e.g., *pro-anorexic beliefs, social/emotional functioning, cognitive rigidity, responses of close others, perfectionism*) reflecting a shared understanding of specific targets for treatment of specific targets for treatment?		0.85	−0.04	0.76
Was the therapist motivational, reflective, client-focused and empathic (e.g., *affirm, emphasize control, support, ask open questions, reflect the client’s comments, ask permission before giving advice, explore the client’s viewpoint*)?		0.84	−0.11	0.81
Did the therapist explore the client’s values and valued domains, and how these have been affected by anorexia, OR refer back to values work as previously introduced?		0.72	−0.03	0.54
Did the therapist encourage the client to externalize their eating disorder (e.g., *through discussions of anorexia as something outside of and separate to themselves*), OR show evidence of externalization through discussions of “the anorexia (or eating disorder, etc)” or “the anorexic voice”?		.72	.06	.59
When questions were used by the therapist, to what extent were they open-ended?		0.66	0.12	0.55
To what extent did the therapist make use of reflections (*simple, double-sided, complex*), and use more reflections than questions?		0.62	−0.16	0.54
Did the therapist refer to previously introduced writing tasks (e.g., *assigned at-home tasks*) or therapeutic letters, OR set writing tasks for between-session work?		0.60	−0.07	0.51
Did the therapist provide feedback on neuropsychological test results and explore with the client how these may relate to eating disorder symptoms, OR discuss links between the client’s thinking style and their eating, behavior or emotions?		0.51	−0.06	0.61
Did the therapist ask the client to report specific thoughts or beliefs (e.g., *dietary rules, thoughts regarding weight or shape)* that the client experienced either in the session or in a situation that occurred prior to the session?	CBT-E	−0.01	0.85	0.71
Did the therapist collaboratively develop a formulation with the client, *making reference to the over-evaluation of eating, weight and shape, strict dietary restraint, mood intolerance, and self-maintaining feedback loops*, which allowed for a shared understanding of the factors maintaining the eating disorder and specific targets for treatment, OR explicitly refer to such a formulation as previously developed?		−0.06	0.83	0.74
Did the therapist encourage the client to record food intake, behaviors and feelings between sessions, OR review the client’s records of food intake, behaviors and feelings from the previous week?		−0.12	0.81	0.72
To what extent did the therapist use Socratic questioning to promote guided discovery (e.g “what does that tell you about…”)?		0.16	0.75	0.58
Did the therapist encourage the client to either (i) view their thoughts as beliefs which may or may not be true, rather than as established facts (e.g., “feeling fat”), OR (ii) use currently available evidence or information (including the client’s prior experiences) to test the validity of the client’s thoughts/beliefs, OR (iii) consider alternative perspectives or viewpoints for events, besides the client’s initial explanations for those events?		0.08	0.70	0.54
Relationship of mood, events and eating: Did the therapist encourage the client to draw links between mood/events and eating, either in relation to past experiences or when discussing future possible experiences?		0.06	0.64	0.41
Did the therapist work with the client to schedule or structure eating behavior/compensatory behavior/exercise?		−0.09	0.56	0.47
Did the therapist work collaboratively with the client to formulate and follow a specific agenda for the session?		0.11	0.55	0.51

**B2:** SWAN-PRS SSCM and non-specific factors items

Item	Subscale
To what extent did the therapist follow the client’s lead in generating issues for discussion?	SSCM
To what extent did the therapist collaboratively generate a list of target symptoms with the client, OR refer back to the target symptom checklist and review functioning in relation to this list?	
To what extent did the therapist give specific advice or suggestions regarding eating or other issues?	
To what extent did the therapist deal with a problem without use of specific Cognitive Behavioural/Cognitive-Interpersonal/Motivational Interviewing techniques?	
Was the therapist empathetic towards the client (i.e., did they convey an intimate understanding of and sensitivity to the client’s experiences and feelings?	Non-specific Factors
Level of Verbal Activity: How much did the therapist talk?	
How much did the therapist direct or guide the session in a subtle way?	
How much rapport was there between the therapist and client (i.e., how well did the therapist and client get along?	
Did the therapist convey warmth?	
How involved (e.g., demonstrating interest, encouraging etc.) was the therapist?	
Did the therapist appear to allow silence to continue (or use minimal encouragement such as “uh huh,” “mm-hmm,” “okay”) as a means of encouraging the client to talk?	
Was the therapist supportive of the client by acknowledging the client’s gains during therapy OR by reassuring the client that gains will be forthcoming?	
Did the therapist actively attempt to engage the client in working together to explore therapeutic issues?	
Summarizing: Did the therapist summarize OR encourage the client to summarize session content of a previous or the current session?	
Did the therapist convey that she understood the client’s problems and is able to help the client?	
How much did the therapist direct or guide the session in an explicit way?	
